# Developing Communication Competency in the Veterinary Curriculum

**DOI:** 10.3390/ani13233668

**Published:** 2023-11-27

**Authors:** Ingrid van Gelderen (Mabin), Rosanne Taylor

**Affiliations:** Sydney School of Veterinary Science, Faculty of Science, The University of Sydney, Camperdown 2006, Australia

**Keywords:** veterinary communication, communication competency, simulated consultations, contextualised scenarios, clinical consultation skills, veterinary curriculum

## Abstract

**Simple Summary:**

Effective communication skills are vital for successful veterinary practice and are a core component of veterinary programs. Veterinary schools design their programs to ensure that all veterinary graduates can demonstrate Day One competencies in clinical communication and provide evidence of this to accrediting bodies. The methods of teaching clinical communication in veterinary medicine have developed since this became a required part of the curriculum over two decades ago, and there is a growing evidence base for their effectiveness. However, validated ‘best practices’ for teaching and assessing ‘real-world’ communication competency are less well established. Here, we report three recent developments in communication skills training in the Doctor of Veterinary Medicine program at the University of Sydney and evaluate implications with respect to curriculum design. The developments are the following: increasing the realism of simulated communications using clinical skills laboratories, embracing a focus on primary care; including telehealth delivery in communications training; and tackling the challenge of “minding the gap” when applying theory to veterinary clinical practice. We conclude that communication in the veterinary curriculum can be more engaging and effective with student-centred design, which increases the realism and authenticity of the student experience.

**Abstract:**

Veterinary graduates require effective clinical communication skills for a successful transition to practice. The ways of teaching and assessing veterinary communication skills have developed and are increasingly supported by research. However, some students have difficulty applying the skills learned in a simulated consultation to working with real clients, particularly in the second part of a standard consultation, where the student communicates the reasons for their clinical decision making and assists the client’s treatment decisions. The authors explore three key developments in communication skills training in the Doctor of Veterinary Medicine program since 2015 at the University of Sydney: (1) Workshops were designed to include communication scenarios that were contextualised in ways that embraced a spectrum of care. These were facilitated within a clinical skills laboratory, and student surveys were used to evaluate this teaching and learning activity; (2) student and facilitator perceptions of the value of online communication skills training were evaluated using surveys; and (3) perceptions of the gap between pre-clinical training and the demonstration of communication competency in authentic clinical settings were evaluated using a survey. We conclude that the communications curriculum can be made more engaging and effective by student-centred design, which increases the realism and authenticity of the student’s experience.

## 1. Introduction

### 1.1. The Importance of Clinical Communication Skills in the Veterinary Curriculum

The teaching and assessment of communication competency are a necessary inclusion in veterinary school curricula. There is a robust body of evidence demonstrating that a successful and safe graduate transition to practice is associated with communication competency development in health professions, including medicine [1] and veterinary science [2,3]. Veterinary schools around the world no longer view communication competency as an optional inclusion or as a set of skills that are learned by example in the curriculum. This is because these skills are amongst the most highly valued and necessary workplace skills for new graduates [4,5,6,7,8,9,10,11,12,13,14,15,16,17,18,19,20,21], and accrediting bodies explicitly require that these competencies be demonstrated by all graduates [22,23,24].

In the packed modern veterinary curriculum, there is a need to explicitly and purposefully embed learning activities and the assessment of communication competency. Explicit communication curriculum frameworks developed from other disciplines such as medicine [25] are widely used as the foundation from which communication skills are taught and assessed in many veterinary curricula [8]. This includes the veterinary curriculum at the University of Sydney, where the skills curriculum has been developed and refined over two decades. With respect to clinical consultation skills (CCSs), the widely accepted framework based on the Guide to the Veterinary Consultation based on the Calgary–Cambridge Model (GVCCCM) [3,8,16] is used to teach and assess CCSs at most veterinary schools in Australia. Clinical consulting skills in a veterinary setting are those behaviours that are demonstrated when undertaking a consultation with a client and include structuring the interaction, developing a rapport and building a relationship, active listening, reflecting, explaining, responding, and concluding with spoken and written interactions. It is expected that the teaching and assessment of CCSs are integrated and sequentially developed within an education program that provides students opportunities to apply these skills in simulated and authentic clinical encounters [26,27,28,29,30]. This is to ensure that students are well prepared to communicate with clients during clinical placements and can demonstrate communication competency at the time of graduation. While the emphasis today is on competency-based education, a review of the medical education literature flags the important link between confidence and competence. With scaffolded experiences and feedback, competency develops over time. As such, activities that enhance students’ confidence contribute to competency development, and, as described in medical education [31], this ultimately helps with a safe transition to professional practice.

### 1.2. Evolving Veterinary Curricula

Veterinary curricula at the University of Sydney have been adapted to address the significant impact of technology and client expectations on the role of veterinarians. This paper describes the changes made to strengthen the scaffolding of students’ communication competency and to smooth their transition to practice.

In the early years of the DVM at the University of Sydney, the focus is on pre-clinical areas of study, and in these pre-clinical years, CCSs are first taught and assessed. CCS training should represent current clinical practice so that students can translate these competencies into a workplace context. This includes the use of clinical skills laboratories where students can practice their skills in safe and structured environments. The curricular revisions described in this paper have focused on strengthening the ways in which students learn CCSs through their experiences, and this was guided by an evaluation of graduate outcomes. Communication skills training plays a significant role in developing veterinarians’ professional identities [32]. As such, course design needs to include resources and environments that afford students authentic opportunities to learn, practice, receive feedback, and be assessed [3,8,10,26,27,33]. Resources such as case studies or scenarios are widely used in different ways in veterinary curricula. This includes their use as a stimulus in workshop discussions, tutorials, problem-based learning activities, and communication skills training. Authentic opportunities to learn and assess CCSs can range from low-fidelity simulations such as role-plays with case scenarios facilitated in a physical classroom (in a clinical skills laboratory or virtually via videoconference) through to immersion in authentic clinical settings where students are supported and supervised whilst applying their communication skills and knowledge in ways expected for a novice veterinarian. The university’s veterinary teaching services are a major site for these authentic environments, where students’ communication skills are then assessed. These experiences are usually in the context of practicing “gold standard” care or a highly specialised approach to veterinary medicine.

A spectrum of care (SoC) approach has recently been recognised as an essential component of practice readiness that requires specific communication strategies [34,35]. To keep pace with industry standards and community expectations, veterinary schools must provide authentic learning activities that reflect a full range of care options, that is, where there is provision and adaptation of veterinary care that suits clients’ preferences, limitations, and relationship with their pet, for example, through shelter medicine or community clinical services [34,35]. With respect to CCSs, veterinary schools are well placed to design case scenarios that reflect complex clinical presentations across a broad range of species and in situations where communication may be challenging. Situated in an environment where research drives the pursuit of new knowledge and innovative practice, highly specialised veterinary teaching hospitals are equipped to scaffold skill and knowledge development that represents a gold standard of care, including advanced diagnostic and therapeutic services. However, veterinary schools must also spend time and resources on the communications skills required for common presentations and routine cases with accreditation bodies in the UK, Australia, and New Zealand, now requiring a substantial focus on common, entry-level practice [22,24]. In practice, the goal of veterinarians is to provide valued services to meet the needs of clients and the animals in their care. So, while offering the best standard of care available to them, their clients will expect to have a range of options communicated to them and receive guidance in the selection of the treatment plan most appropriate to their needs. Clients may experience significant financial or practical constraints or simply have different priorities in what health care they are able to support for their animals [34,36,37]. As such, it is important for veterinary schools to include the teaching and assessment of communication techniques that help the veterinarian navigate this SoC approach.

Opportunities to apply clinical knowledge and skills in a spectrum of authentic professional practice environments are a critical component of veterinary education programs, and these are most effective when they build students autonomy and decision making in a supported environment. These holistic experiences afford students the opportunity to integrate communication skills with the full range of technical and non-technical competencies expected of a veterinarian. Veterinary schools achieve this in a variety of ways, including the involvement of veterinary teaching hospitals within the university in contracted practices as well as supervised practice with external partners in the industry. This presents a range of challenges, one of which is ensuring that there is vertical alignment between formal teaching and assessment activities and what is then subsequently expected of students when they apply their skills in authentic clinical encounters. Clinical placements in the final years of a veterinary program are recognised to be an effective way to link theory to practice [38]. However, research investigating work-integrated learning environments in other disciplines (health, education, psychology, and law) has shown that aligning authentic assessment activities in this complex context is challenging and problematic [39]. For example, when confronted with an actual clinical case in veterinary practice, information is often incomplete, or the veterinarian is presented with a client that has limited funds or does not have the capacity to understand or adhere to the recommended therapeutic plan. Communication with clients is impacted by the student’s ability to manage time, reach probabilistic decisions, and explain these as they guide the client’s treatment choices. How students experience the practice of communication skills in clinical placements and how well clinical teachers’ and supervisors’ assessment and feedback align with underpinning theoretical frameworks such as the GVCCCM is not well understood and remains an ongoing challenge with respect to consistency in feedback and assessment by clinical teachers and supervisors.

The challenge for veterinary schools is to make sure that curriculum review and renewal reflect industry and community expectations. Over the past 30 years, innovations in the way we communicate have been phenomenal: the general public has access to a vast amount of information via the internet, and they bring this with them to the consultation room; the widespread use of social media impacts the veterinarian–client relationship in varying ways; the role, skills, and daily work of veterinarians are much better understood due to extensive exposure through documentaries on television; telehealth is now widely practiced in human health; and the use of technology has revolutionised the way in which veterinarians record their consultations and communicate with their clients. In sum, clinical communication skills are not static competencies. This was starkly illustrated during the recent COVID-19 pandemic. As universities and veterinary schools grappled with the required shift to online teaching and learning, the same social distancing requirements led to the rapid industry uptake of telehealth consultations. That is, as the teaching and assessment of CCSs moved online, veterinarians were moving to telehealth consultations. This presented a unique opportunity to explore and review the way in which many aspects of the veterinary curriculum were taught and assessed, both in terms of educational value and industry currency.

### 1.3. Curriculum Progression of CCSs at the University of Sydney

Since 2015, at the University of Sydney, CCSs have been formally taught and assessed in the first and second years of the DVM program of study (DVM1 and DVM2). Opportunities to practice, demonstrate competency, and be assessed on these skills are subsequently provided in community spey-neuter clinics in the third year of the DVM (DVM3), in teaching hospitals, and in external placement rotations of final year (DVM4). The formal teaching of CCSs is first integrated into a professional practice Unit of Study (UoS), and this is followed by a series of small workshops in a module that sits within a larger, first-year Professional Skills UoS in the DVM program. Workshops are conducted throughout the first semester in DVM1 and the second semester in DVM2.

In the first year of the DVM program, the formal clinical communication curriculum involves an introductory lecture and a large-group workshop that is then followed by a series of small-group workshops (5–6 students per group). The small-group workshops are facilitated by veterinarians who have recent or current clinical experience. It is intended that the introductory lecture on clinical communication focus on understanding the generic skills required for effective communication as well as describing models for effective clinical consultations. The first large-group workshops (20–24 students per group) take place after the introductory lecture and involve several activities and group discussions. These explore elements of the clinical consultation framework and include activities that examine specific communication skills such as empathy, non-verbal communication, and the use of proxemics when communicating in a consultation. The subsequent small-group workshops involve students conducting simulated consultations as both a veterinarian and a client for a range of common companion animal health issues that include advice on desexing procedures, weight management recommendations, dental care, guidance on rabbit husbandry, and an equine health issue. Students conduct the simulated consultations for a standardised series of cases, and during the workshops, they reflect on their achievement against their own communication SMART goals, that is, goals that are Specific, Measurable, Action-oriented, Realistic, and Time-limited [40]. Students then receive structured feedback from their peers and their facilitator using the GVCCCM framework. At the end of the workshop series, students are allocated an assessment case from one of the health issues that they have been introduced to, and they conduct a simulated consultation with a professional actor taking on the role of a client. The facilitator then assesses student competency based on the core clinical consultation skills that are mapped to the GVCCCM.

In the second year of the DVM program, students’ second professional practice UoS has a focus on some more challenging elements associated with clinical consultations. Students then practice their communication skills and receive feedback in a series of workshops that spotlight more challenging clinical consultations. In these workshops, the standardised cases now focus on issues such as revisit, euthanasia, emergencies, and discharge consultations for complex cases. Hospital teaching placements during Professional Skills UoS in DVM1 and DVM2, pre-clinical placements in DVM3, the community spey-neuter clinic in DVM3, and the final year placements provide students with opportunities to apply their CCSs in authentic clinical encounters that vary based on species and context.

At the University of Sydney, Units of Study with explicit learning outcomes and assessment activities associated with clinical communication skills are intentionally mapped across all stages of the DVM (Table 1). This table shows how these Units of Study are sequentially developed at the University of Sydney, with underpinning knowledge of CCS frameworks explored in the professional practice Units of Study in DVM and opportunities for the application of CCSs at the pre-novice stages in the Professional Skills Units of Study, industry, and pre-clinical placements. Context- and species-specific clinical communication learning outcomes are mapped across all DVM3 Units of Study, along with the experience of leading a community spey-neuter consultation and discharge. During the final clinical DVM4 year, learning outcomes that align with communication skills competency are articulated in all final year placements. Students are scaffolded with increasing levels of complexity and challenge as they progress through the DVM program and move from the pre-clinical to the clinical curriculum in DVM4. The authors submit that CCS training should be introduced early in the curriculum and progressively developed in challenge and complexity across the years.

This article explores the dynamic evolution of CCS education in veterinary medicine since its inception in the DVM curriculum at the University of Sydney. Three key developments are explored, and their implications are critically evaluated. These developments include:

1. Enhancing contextualisation through skills laboratories: a close examination of CCS sessions in DVM1 and DVM2, embracing the spectrum of care approach.
2. Navigating the era of online CCSs: analysing the impact of COVID-19 restrictions on CCSs, with insights into telehealth and future pedagogical implications.
3. Bridging theory and practice: an in-depth evaluation of the application of CCS training in authentic clinical environments throughout the DVM program, tackling the challenge of ‘minding the gap’.

## 2. Materials and Methods

### 2.1. Utilising Skills Laboratories—Enhancing the Spectrum of Care Approach

In 2017, a pilot was conducted in which technical skills typically conducted in a consultation were incorporated into the CCS module for DVM students. DVM students engage in a series of CCS workshops in DVM1 (CCS1-6) and then DVM2 (CCS7-10). A single CCS session was dedicated to performing a “bed-side” selection of routine tasks such as calculating drug doses, blood collection, and routine diagnostics while students undertake four simulated consultations. Several skills were embedded within each simulated consultation, but they were skills that are performed by veterinarians within the time constraints of a typical consultation. The pilot focused on a single DVM2 workshop session, as students in this cohort had developed and practiced a greater range of routine technical skills at this stage of their study program. Consultation with other staff coordinating professional skills guided the CCS coordinators in aligning this session with the curriculum in DVM1 and DVM2. Facilitators were trained in a short workshop, had access to online readings and additional preparatory materials for each class, and then reflected on their experiences with the coordinator.

Groups of 5–6 students and one facilitator rotated through four “bed-side” stations within a clinical skills laboratory (CSL), also referred to as a clinical skills space at the Sydney School of Veterinary Science (and previously named the clinical skills hub). This space is a large, flat-floored teaching room equipped with benches for groups of up to 6 students per bench and a range of equipment for common veterinary procedures conducted in a routine consultation. Each station was conducted around a common clinical scenario. Students were given a brief case history, a list of tasks to perform, and the skill set that was required to manage the case. Instructions for each task were provided in the form of step-by-step procedure guides, images, and short videos where relevant. These instructions were available on the Learning Management Platform via an iPad at each station and were available in hard copy during the workshop. A series of questions and prompts were provided to facilitators to initiate discussion amongst students about the role of communication in the management of each case. Students were on their feet and were required to make decisions, perform the procedures, and role-play communication with their clients and team members as they proceeded through the cases. The time spent on the role-play was purposely limited to 10 min to encourage time management. The four cases were structured around common clinical scenarios that are routinely seen in veterinary clinical practice (Table 2).

Anonymous student feedback evaluating the value of this teaching and learning experience was gathered via an online student survey administered using Survey Monkey^®^ (basic). This was a reflective teaching evaluation activity, and no statistical analysis was performed on the data. There were 84 responses from a cohort of 120 students.

### 2.2. Online Teaching and Assessment of Clinical Consultation Skills

In response to the COVID-19 restrictions regarding social distancing, CCS workshops for DVM1 and DVM2 students moved to online delivery (using Zoom) and assessment in 2020 and 2021. Offshore and onshore students were located across three continents and multiple time zones. Many key elements in the administration of CCS workshops remained the same. The case-scenario health issues were the same as those used for in-person delivery, and groups of 4–6 students participated in a series of workshops that were facilitated by a veterinarian with recent or current clinical experience. Once all workshops were completed, students were individually assessed by a facilitator, and the ‘client’ was a professional actor. Students were given feedback and assessed on the same core communication skills as those used for in-person delivery. These included preparing and initiating the consultation; exploring the patient’s history; using attentive and reflective listening strategies; demonstrating effective explanation and planning; closing the consultation; providing structure to the consultation; employing effective non-verbal communication; expressing empathy; seeking and receiving constructive feedback; and structuring and providing constructive feedback for peers. 

Some changes to the teaching and assessment of CCSs were required in response to the need for social distancing and the COVID-19 restrictions. Prior to the first two workshops in the COVID-19 lockdown period, students were required to submit a video of themselves conducting a simulated consultation (adhering to isolation requirements). This video was uploaded to a discussion page on the Learning Management Platform, Canvas. For the remaining CCS sessions, simulated consultations were conducted live during the Zoom workshops. Small-group, facilitator-led workshops were all conducted via Zoom and involved student goal setting, reflection on the consultations, and constructive feedback shared by peers and the facilitator. The final workshop and the assessment involved students conducting a consultation using existing cases that were modified to suit a telehealth format, and this was based on the Australian Veterinary Association telemedicine guidelines released in response to the COVID-19 restrictions. In the workshop, the role of ‘client’ was filled by a fellow student, and for the assessment, a professional actor was used. Constructive feedback was provided by peers and facilitators during the workshop, and for the assessment, student competency was determined by the facilitator with respect to the core communication skills that are mapped to the elemental stages in the Guide to the Veterinary Consultation based on the Calgary–Cambridge Model (GVCCCM) [8].

As reported in van Gelderen et al. [41], an online, anonymous survey evaluating the student, facilitator, and actor experience was administered at the conclusion of the program, with data collected via the secure data collection instrument Qualtrics^®^. The survey was administered to all DVM1 students (119) and facilitators and actors (14), with 20 completed survey responses received from students and 10 from facilitators and actors. Facilitators who were involved in collecting and analysing the data were not included. The survey questions were designed to evaluate four key areas:

Value of the learning experience

Level of confidence

Perceived ability

Emotional response

Questions had five response options using a Likert scale from ‘strongly disagree’ to ‘strongly agree’, and an optional open-text response box was available to gather additional comments for each question. Questions for the facilitators and actors were modified to reflect their perspective and to seek comparative data as this group of participants had prior experience with CCSs offered in person [41].

For each of the four key areas under investigation, the levels of agreement (strongly agree and agree) were measured for the participant groups. Data comparing the online experience to the in-person CCS experience were only collected from the facilitators and actors. Free-text responses to the open-ended questions were reviewed by all researchers, with the lead researcher initially developing key themes through keyword searches and pattern identification. Repeated discussions amongst the research group addressed biases, and a final set of themes was established based on a concordance of views from the three researchers [41] (see https://library.iated.org/view/VANGELDEREN2021FRO (accessed on 23 November 2023)).

Ethics approval for this study was granted by the University of Sydney’s Human Research Ethics Committee, Project Number 2020/496.

### 2.3. Evaluating the Application of CCSs in Authentic Clinical Settings

In 2022, a survey was designed to evaluate the effect of clinical communication skills training on supporting DVM students’ application of CCSs in authentic clinical encounters. The study sought to evaluate students’ perceptions of their competency in communication skills, their confidence levels, and whether training supported their application in an authentic environment. Due to pandemic isolation impacts, these students had varying levels of opportunity to apply communication skills in authentic settings, so the survey included questions to establish the students’ stage of learning and level of opportunity.

An online anonymous survey instrument was administered to all DVM students at the University of Sydney who had completed formal CCS training in DVM1 and DVM2, and survey questions were modelled on a study designed to track the acquisition of CCSs and student confidence following training using the GVCCCM framework [29]. This survey was anonymous and was administered after all teaching and learning and formal assessments of CCSs were complete. Qualtrics^®^, a secure survey data collection instrument, was used for survey administration.

General descriptive data were gathered from students regarding the stage of the degree that they were in, the number of opportunities that they had experienced to apply their communication skills, where they had applied these skills, confidence levels for different types of skills, and capability levels for different types of skills. Questions exploring students’ confidence level in individual communication skills included response options on a five-point Likert scale from ‘lack confidence completely’ to ‘completely confident.’ Additional questions included a yes–no–unsure assessment of the effectiveness of clinical communication skill training and a self-assessment of the ability to build rapport, take a comprehensive history, and make a client feel valued. 

Survey data were collected over 12 weeks, and 35 valid responses from the 272 students invited to participate were received. The data analysis report was downloaded from Qualtrics^®^, and raw data were then cleaned for analysis. The data were analysed using GenStat^®^ using a logistic regression model with an underlying binomial distribution. This analysis sought to identify if there was a statistical association between the outcomes (building rapport, taking a history, making a client feel valued) and stage of degree and the number of opportunities the students had to apply their skills.

Ethics approval for this study was granted by the University of Sydney’s Human Research Ethics Committee, Project Number 2022/058.

## 3. Results

### 3.1. Utilising Skills Laboratories—Enhancing the Spectrum of Care Approach

The informal survey evaluating the utilisation of the clinical skills space for a single session in the CCS workshop series revealed that the majority of students either strongly agreed or agreed (96%) that this session helped their learning. Most students (93%) could understand the link between the skills performed and the impact on communication during a consultation, and 96% of respondents believed that this session helped their learning of clinical communication. The areas identified for improvement were associated with logistics, namely the organisation of the stations in the space, the quality of the supporting materials for students, and the scaffolds for facilitators and students (Table 3).

In response to the student feedback received, the module coordinator developed and incorporated a similarly designed session into the series of DVM1 CCS workshops in 2022. The DVM1 sessions in the CSL focused on simple cases and skills that aligned with this stage of the curriculum (Table 4). More emphasis was placed on facilitator prompts, guidance, just-in-time feedback, and short role-plays to promote the discussion of communication skills so that students could clearly identify the links between the performance of these ‘bed-side’ skills and their impact on communication skills. These were provided to facilitators to improve the consistency and organisation of activities in the clinical skills space. Additional prompts were included to highlight issues related to cultural competency.

### 3.2. Online Teaching and Assessment of Clinical Consultation Skills

In evaluating online teaching and assessment of CCSs, participant responses were grouped according to four key areas (Table 5). High levels of agreement (>90%) were identified from the student and facilitator/actor groups with respect to the ‘value of the learning experience’ and the ‘increasing ability to communicate effectively’. Facilitators and actors reported lower levels of agreement than students for ‘increasing confidence to communicate in a veterinary consultation’, ‘dealing with clients’, and ‘encouraged active participation’. Students reported higher levels of ‘increased ability to structure a consult based on GVCCCM’ compared to facilitators and actors. Most facilitators and actors (70%) reported that online CCS teaching and assessment were not more effective than in-person delivery.

There were six themes identified from the qualitative survey data describing the participants’ experiences of online CCS teaching and assessment. Consistent themes were identified in both the student (ST) and facilitator (FA) groups, with some variation in emphasis and focus within each theme. See Table 6 for themes, key points, and illustrative quotes.

### 3.3. Evaluating the Application of CCSs in Authentic Clinical Settings

**Figure 1:**
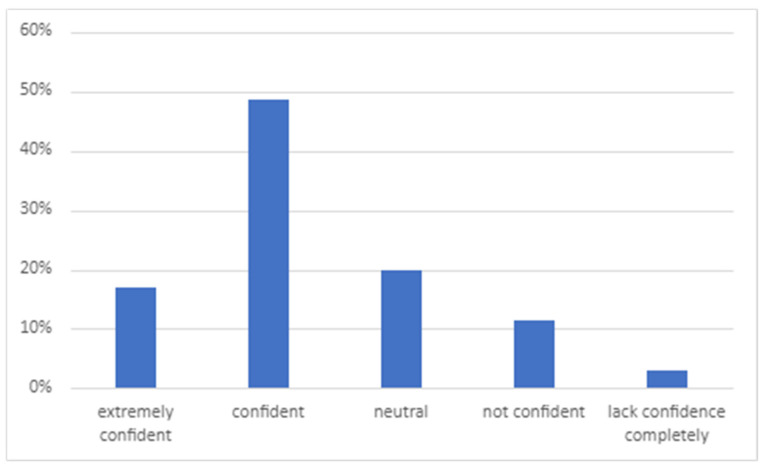
Students’ perceptions of overall confidence in clinical consultation skills (*n* = 35).

**Figure 2:**
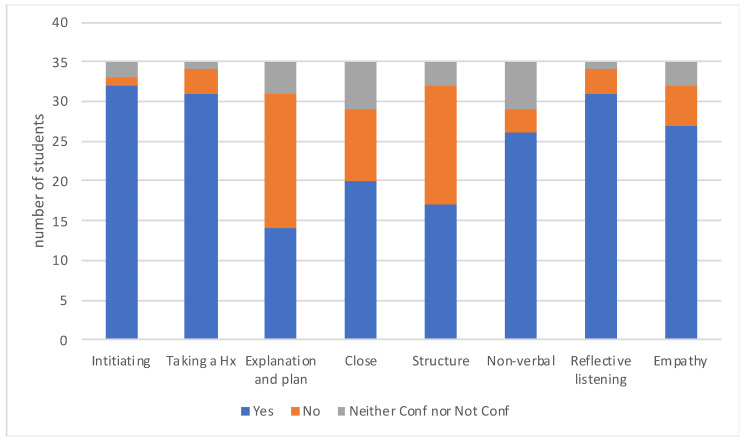
Students’ perceptions of confidence in specific clinical consultation skills (*n* = 35).

**Figure 3:**
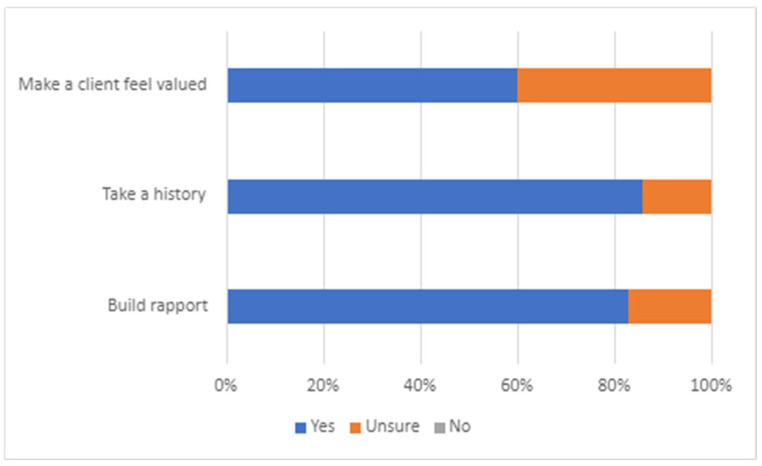
Students’ perceptions of competency in specific clinical consultation skills (*n* = 35).

**Figure 4:**
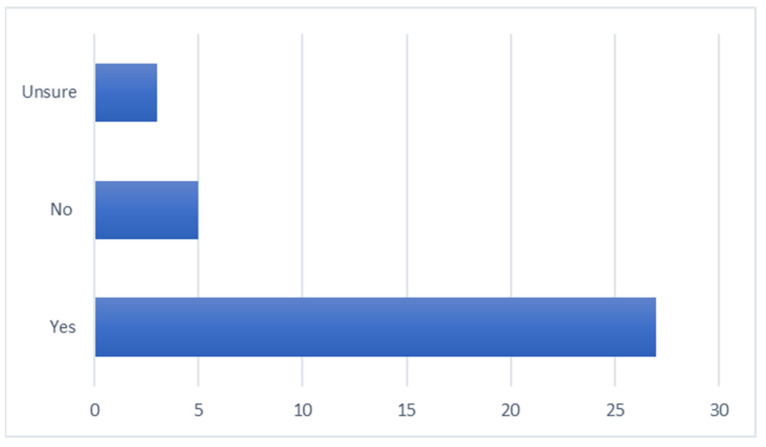
Students’ evaluation of whether CCS training in DVM1 and DVM2 supported application in an authentic clinical encounter (x-axis is the number of students and *n* = 35).

The data obtained from the survey (response rate 13%) evaluating the students’ application of CCSs in an authentic environment revealed information about the opportunities that students had to apply CCSs, the overall confidence levels in CCSs (Figure 1), the confidence levels in specific skills (Figure 2), the perceived competence in taking a comprehensive history, the building of client rapport and making a client feel valued (Figure 3), and an evaluation of whether CCS training supported the application of skills in an authentic clinical encounter (Figure 4). The respondents were at varying stages of their degree. Those at the early stage (43%) had completed their CCS training workshops in DVM1 and DVM2 and had just commenced DVM3. Most of these early-stage students (70–80%) had completed their two 2-week pre-clinical placements in university-operated or private veterinary clinics. The remaining respondents were either in the mid stage (49%), having completed DVM3; their pre-clinical placements and one or two final year rotations; or their late stage (9%), where they had completed all final year rotations in DVM4 and had just recently graduated.

More than half of respondents reported having had more than 10 opportunities to apply CCSs in an authentic clinical setting, and the highest number of opportunities were undertaken during pre-clinical placements. The ‘other’ opportunities were all linked to external work as a veterinary nurse or technician. The majority of respondents (77%) reported that formal training in CCSs supported their skill development (Figure 4). Most respondents (66%) were confident or extremely confident in applying CCSs, 20% were neutral, and 14% were not confident or lacked confidence completely. Statistically, there was no significant association between confidence levels and the stage that respondents were up to in their degree (Table 7). However, an analysis revealed that those respondents who had more opportunities to apply their skills were more likely to report being confident or extremely confident.

The specific CCS skills based on the GVCCCM framework where students reported the highest confidence were ‘initiating a consultation’, ‘taking a history’, and ‘reflective listening’ (Figure 2). Those CCS skills where students reported the lowest confidence were ‘explanation and planning’, ‘providing a structure’, and ‘closing the consultation’. With respect to students’ perceptions of their competence in CCSs, it was revealed that most respondents were certain that they could build rapport with a client and take a comprehensive history, but there were higher levels of uncertainty when asked about making a client feel valued (Figure 3). Statistically, there was no significant effect of the stage of degree on any of the questions relating to perceived competence, and the only significant association was related to the number of opportunities that a student had to apply their skills in an authentic clinical setting and make a client feel valued (Table 7). That is, those respondents who had more than 10 opportunities to apply their CCSs in an authentic clinical setting were nine times more likely to perceive that they were competent in making a client feel valued (95% CI 1.8–44.8) (Table 8).

## 4. Discussion

A spectrum of care (SoC) approach is recommended in veterinary education [35,36,37], and there are many ways that this can be successfully achieved across a veterinary curriculum with respect to communication competence. Veterinary graduates are expected to have competence across multiple domains and be able to adapt in response to the diverse needs of their clients and society. Competency-based veterinary education (CVBE), as described by the Association of American Veterinary Colleges, describes nine domains of expected competence necessary for veterinary graduates [42]. Suggested subcompetencies within the Communication Domain explicitly address the fact that clients and society have varied needs, wants, and understandings regarding the health of animals. Clinical consultation case studies or scenarios are a widely used curriculum resource with respect to communication skills training and assessment, and these scenarios are easily adapted to demonstrate a wide range of different outcomes with respect to patient care. The capacity to tweak and modify case scenarios in real time was a key feature identified in the CCS workshops that were undertaken in the clinical skills space at the University of Sydney. Moreover, the scenarios were designed with consideration of time management with respect to making decisions on clinical information and communicating that to the client, making these simulations a valuable tool for assessment and feedback. Facilitators were able to prompt students to consider how they would need to adapt communication styles as well as diagnostic and therapeutic plans because of varied client understandings, needs, wants, and financial capacities.

Training and guidelines for facilitators and teachers are necessary to achieve the communication competency outcomes described here [6,8,25]. Facilitators of CCS workshops at the University of Sydney are practitioners who have recent or current experience in veterinary practice, and they are well placed in their understanding of common scenarios and the variation in what clients understand, need, and want with respect to the care of their animals and their capacity to support that care. It is, however, critical that facilitators are active in promoting these discussions with their students. As such, facilitator training and supporting guidelines with suggested prompts are required to support the standardised case scenarios used in CCS training and assessment. Moreover, guidance to facilitators and students on providing and receiving effective feedback is needed to reinforce, encourage, and scaffold [41].

Simulations can greatly assist in preparing students for the responsibility of case management [43]. Learning through the experience of being responsible for case management is an essential aspect of students’ final rotations during veterinary school, and it is critical when they transition into clinical veterinary practice. Indeed, the transition to taking responsibility for decisions and communicating with others is a difficult transition for many students, challenging their confidence and self-perceptions of competence. This is true for both technical skills and non-technical skills, such as clinical communication. Case management involves communication that does more than inform clients about diagnostic and therapeutic plans. When communicating with clients, veterinarians guide clients in the decision-making process, and they need to help their clients understand the implications of the choices made. This includes, but is not limited to, consideration of finances, understanding cultural differences, and client constraints. Simulated consultations, embedded early in the veterinary curriculum, are effective in providing students with repeated opportunities in a safe environment to practice and finesse those communication skills that are required for effective case management. This includes experiencing the impact of client ‘pushback’ or negative emotions in response to veterinary advice, navigating and negotiating diagnostic and therapeutic plans, and managing time in a consultation. According to the study reported in this paper, most students reported that the formal integration of simulations was effective in supporting their development of clinical communication skill competency. Using the GVCCCM framework to interrogate this further, it is evident that CCS training supported students’ confidence and competence in gathering a history, active listening, and building a rapport with their client, but there was less certainty in students’ confidence for making a client feel valued. Significantly, students who had more opportunities to apply their communication skills in an authentic clinical setting reported higher confidence levels in making a client feel valued. Providing context is key to the assessment of communication skills [44], and it is not surprising that authenticity in the design of learning experiences is critical to enhancing student confidence.

Communication skills cannot simply be viewed as individual competencies. In research investigating the factors contributing to complaints and litigation in the veterinary profession [20], communication issues played a role in 80% of cases. This means that when we teach and assess communication skills, consideration must be given to organisational systems and communication within teams. As such, this must underpin the design of simulations (case studies, facilitator prompts, environment, distractions, etc.) that are used in communication skills training. For example, when using the clinical skills space for the session when communication skills were contextualised, students were time-limited when conducting the role-plays whilst performing routine skills expected during a consultation (for example, administering a tablet to a fractious cat owned by an elderly and frail client). This challenged the student to consider how they would communicate with the client and their team to ensure that the consultation was completed in a timely way whilst at the same time achieving the desired outcome, maintaining the client’s trust, and maximising the chance of client adherence to the therapeutic plan.

There are aspects of clinical communication competency that are likely to present greater challenges to the veterinary student and will be more difficult to teach and assess in the veterinary curriculum. This is particularly the case in the early stages of the DVM when students have little or varied experience and their knowledge is still foundational. As such, their clinical reasoning skills and their capacity to make decisions and take responsibility for those decisions are limited. Clinical reasoning relies on the ability to prioritise problems and then take a systematic and reflective approach to investigate and take action on those plans [3,45]. Even a reliance on the risky practice of pattern recognition using a non-analytical form of reasoning will pose challenges for the novice with limited experience of common presentations. It is therefore unsurprising that students report lower levels of confidence when structuring a consultation, providing their client with an explanation and plan, and closing the consultation. That is, when they must integrate interpersonal communication with clinical decision making. This is supported by previous research that found students demonstrate lower levels of competence with organisation and sequencing [28], and in Meehan and Menniti’s study [46], students found it more difficult to manage the consultation with respect to time, structure, and discussions of costs. This is likely to be amplified when problems are complex and there are higher levels of uncertainty. In real time and with a foundational knowledge base, it will be more challenging for students to prioritise information, design, and then action plans in collaboration with their clients.

In this paper, we have described an integrated approach in which students are given explicit opportunities to apply communication skills using simulations that utilise clinical skills laboratories in the pre-clinical stages of the degree and then in actual consultations in their final year placements. Clinical skills laboratories are used in many veterinary schools for the provision of self-directed learning opportunities and the formal teaching of technical skills prior to performing the skills in a live setting (see Baillie et al. [47]). This paper reports on the use of a CSL in the teaching and assessment of communication skills, and, whilst this is not a widely reported use of a CSL, it was raised in some of the survey feedback reported in Baillie et al.’s study [47]. For the use of a CSL to work in communication skills training, the authors suggest that case scenarios be carefully designed so any inclusion of “bed-side” technical skills aligns with what students would already know and would be able to perform at the stage of the curriculum when communications skills workshops take place. Time-bound simulations where students experience performing a range of routine skills, such as dose calculations for medications, whilst communicating with their client are a way to help students practice their communication skills in a safe, low-stakes environment. The role-plays conducted for the CSL pilot were limited to 10 min to encourage time management, and this feature was incorporated into the design of subsequent CSL workshops in DVM1 and DVM2. The authors recommend that the emphasis on facilitator scaffolding and feedback remain focused on communication skills, and this needs to be reinforced in facilitator training. Prompts that can be used to tweak the scenario to represent the spectrum of care options need to be provided to facilitators and reinforced with training. Small student groups are therefore essential so that students are able to practice and receive meaningful feedback. This aligns well with the core components of a competency-based veterinary education curriculum, in which learning outcomes can be progressively sequenced across the program of study [42].

When veterinary educators review a veterinary curriculum in the context of competency-based education, the teaching and assessment of communication skills can be enhanced by explicitly describing observable markers, or milestones, that define the level of skill for each competency in the Communication Domain in the pre-clinical (pre-novice) stages and then from novice to advanced beginner, competent, and proficient in the clinical rotations in the final year. In the final placement year, a way to link communication competency to application in a professional, authentic workplace may be through a defined Entrustable Professional Activity (EPA). The AAVMC CBVE has described core EPAs [42], and the authors posit that the integrated and authentic context for learning and the communication skills feedback provided by an EPA may go some way to enhancing students’ confidence in the face of uncertainty and complexity that is typically faced in practice. The authors acknowledge that the small sample sizes of students participating in the surveys are a limitation for the results reported in this paper. However, we maintain that monitoring the student’s experience across the program is necessary to assure the integrity of communication skill training, and this may be achieved through student and graduate surveys, focus interviews, and supervisor feedback reports.

Finally, it is worth noting that recent innovations in the way that professionals communicate in practice should be given some consideration with respect to future curriculum planning. By way of example, the impacts of the COVID-19 pandemic amplified the use of communication methods that use technology, and this was demonstrated in the widespread endorsement of online teaching and learning in higher education [41] and the increased uptake in telehealth services by veterinary practitioners [48,49]. Prior to this, veterinarians in practice had not widely embraced telehealth options [49], and, to the authors’ knowledge, there is no evidence that these methods of communicating are widely embedded in communication skills training in veterinary schools. The survey reported in this paper indicates that facilitators and some students recognised the value of the online CCS training implemented during the pandemic as it aligned with what practitioners were required to embrace at the height of the pandemic restrictions [41]. As new ways of accessing and communicating information continue to develop at an astounding pace (see Aliwi et al. [50] for a review of the use of augmented and virtual reality technologies in medical communication), it is likely that the practice of veterinary telemedicine or telehealth will increase [49,51]. As such, it behoves educators to consider how this can be addressed in the teaching and assessment of communication skills in the curriculum, which is an area for future research.

## 5. Conclusions

Mastering effective communication skills is vital in veterinary education. Public trust, accreditation mandates, and research-backed methodologies converge to emphasise the essential role communication skills play in ensuring optimal animal welfare outcomes, making them a cornerstone of veterinary training. However, validated ‘best practices’ for teaching and assessing ‘real-world’ communication competency are not well established [12,18]. The authors of this paper present several strategies to develop communication skills training in a veterinary curriculum and also suggest areas that may be considered for future research. These strategies enhance the ways in which CCS training and assessment are contextualised. Clinical skills laboratories can provide a more authentic, enriched, and engaging learning experience and introduce students to communications with clients around selecting patient care options from the spectrum available. Students can be better prepared for telehealth consultations by incorporating these into clinical communications training. Finally, veterinary schools should monitor the cohesion and integration of the communication elements in the curriculum and understand students’ experiences and graduates’ perceptions of their confidence as well as competence to ensure that all veterinary program graduates are ready for a smooth transition to practice. A dynamic and ever-evolving curriculum is essential, and our proposed strategies for developing communication competency offer practical ways for veterinary schools to ensure graduates are not only well prepared for current challenges but also equipped for the evolving demands of the future.

## Figures and Tables

**Table 1:** Units of Study with explicit communication learning outcomes and assessments in the DVM program at the University of Sydney.

	Professional Practice	Professional Skills	Placements
DVM1	VETS6101: The Veterinary Professional 1	VETS6102: Professional Skills 1A VETS6106: Professional Skills 1B	
			VETS6121–VETS6128: Industry Placements These placements occur at farms and other commercial animal enterprises for different animal species.
DVM2	VETS6201: The Veterinary Professional 2	VETS6202: Professional Skills 2A VETS6206: Professional Skills 2B	VETS6121–VETS6128: Industry Placements
			VETS6221–VETS6222: Preparatory Clinical Placement
DVM3	VETS6301–VETS6314: VETS6308: Veterinary Practice Management: Professional Practice Unit of Study VETS6314: Small Animal Desexing Clinic All other DVM3 units include clinical communication learning outcomes that are context- and species-specific.		VETS6221–VETS6222: Preparatory Clinical Placement
DVM4			VETS6401–VETS6404: Small Animal Clinics VETS6405–VETS6406: Large Animal Clinics VETS6408: Public, Industry or Community Placement VETS6409–VETS6412: Extra-mural Placements

**Table 2:** Pilot DVM2 communication workshops in the CSL: case scenarios and technical and communication skills.

Case	Technical Skills	Focused Communication Skills
Clinical consultation room—first-time client with new puppy	Using clinic software (Rx Works—demonstration version) to gather information when taking a history and advising on diet, training, and behaviour.	Gathering appropriate amount and type of information and managing proxemics in the consultation room.
Trauma case—hit by car	Dose calculation, placing IV catheter, calculating fluid rates, wound care, bandaging, and completing drug register for controlled drugs.	Evaluating barriers to effective communication and expressing empathy (owner was the driver and incident witnessed by children).
Revisit case—fractious cat with elderly client	Administering subcutaneous drugs, venepuncture, and performing routine blood tests (PCV, TPP), cystocentesis, and urinalysis.	Evaluating barriers to effective communication, understanding client perspective, empathy, structure of explanation and plan, and chunk-and-check techniques.
Rescue case—sick puppy presented by foster carer	Basic clinical pathology such as faecal float, heartworm test, fine-needle aspirate, and dose calculation.	Evaluating barriers to effective communication, gathering history when information is incomplete, and communicating complex therapeutic plans in situations that are complex with barriers to adherence.

**Table 3:** Questions and survey responses from DVM2 students evaluating a CCS workshop in the CSL (clinical skill hub), *n* = 84.

Questions	Strongly Agree	Agree	Neutral	Disagree	Strongly Disagree
Q1: Working on skills commonly encountered during a consultation in the clinical skills hub helped my learning	71%	25%	1%	1%	1%
Q2: I understood the link between the skills performed and the impact that this has on communication in a consultation	62%	31%	5%	1%	1%
Q3: The stations in the clinical skills hub were well organised	43%	51%	4%	2%	0%
Q4: I had enough time to work through the skills at each station	19%	37%	19%	20%	5%
Q5: The supporting videos and information material on the Learning Management Platform helped me to prepare for the session in the clinical skills hub	36%	44%	19%	1%	0%
Q6: The discussion questions for each scenario helped my learning	46%	43%	8%	1%	1%
Q7: Overall the CCS clinical skills hub session helped my learning of clinical communication	55%	36%	6%	2%	1%
Q8: Overall the CCS clinical skills hub session helped me to develop skills I will need for success as a veterinarian	70%	26%	1%	1%	1%

**Table 4:** DVM1 communication workshops in the CSL (clinical skills hub): case scenarios and technical and communication skills.

Case	Technical Skills	Focused Communication Skills
Obese dog with osteoarthritis	Dose calculation, administration of subcutaneous injection, and interpretation of feeding instructions	Structuring an explanation and plan, achieving shared understanding, expressing empathy, and cultural competency (owner is elderly, hearing and vision impaired, and cannot read the feeding instructions on the bag of dog food)
Cat abscess and castration advice	Dose calculation, writing drug labels, interpreting in-house rapid blood tests (e.g., FIV/FeLV SNAP test), and oral tablet administration	Structuring an explanation and plan, expressing empathy, negotiating a therapeutic plan, achieving shared understanding, and cultural competency (owner is resistant to castration based on prior experiences and cultural norms)
Dog with sore ears, retained deciduous teeth, and ovariohysterectomy advice	Use of equipment (otoscope), basic ear cleaning, and use of anatomical models to demonstrate normal and pathologies, e.g., those observed in canine ears and teeth	Structuring an explanation and plan, expressing empathy, negotiating a therapeutic plan, achieving shared understanding, closing the consultation, and discussion of costs
Annual health check and prophylactic care (vaccination and internal and external parasite control)	Dose calculation, microchip scanning, vaccination card completion, review, and selection of appropriate parasiticide product	Questioning techniques, signposting techniques, reflective listening, structuring an explanation and plan, negotiating a plan with shared understanding, and closing the consultation

**Table 5:** Levels of agreement evaluating four key aspects of the online CCS teaching and learning experience for students (*n* = 20) and facilitators/actors (*n* = 10) (taken from van Gelderen et al., 2021 [41]).

	Students: Proportion Reporting Strongly Agree and Agree (%)	Facilitators/Actors: Proportion Reporting Strongly Agree and Agree (%)
Value of learning experience		
Was a valuable learning experience	95	100
Effective in developing communication skills	95	100
Effective in the integration of clinical knowledge and communication skills	100	90
Was more valuable than face-to-face	N/A	10
Confidence		
Increased confidence to communicate in a veterinary consultation	90	70
Increased confidence to deal with clients	80	60
More effective than face-to-face CCSs in developing confidence	N/A	30
Ability		
Increased ability to communicate effectively for common scenario	95	90
Increased ability to structure consult based on GVCCM	100	80
More effective than face-to-face	N/A	20
Emotional response		
Was a stressful experience	20	30
Encouraged active participation	95	80
More stressful than face-to-face	N/A	0

**Table 6:** Participants’ experiences of online CCSs: emergent themes and illustrative quotes from 197 comments (94 comments from 10 facilitators/actors (FAs) and 103 comments from 20 students (STs)) (taken from van Gelderen et al., 2021 [41]).

Theme	Key Points	Illustrative Quotes
1. Online teaching and learning	Benefits associated with online formats, such as more time to prepare video uploads, which reduced stress. Online had inherent limitations but was an authentic representation of the shift to telehealth	*[online] less confronting for students and promoted discussion* (FA) *[online] not a full substitute for some physical consultations, different feel* (ST)
2. Skills and knowledge	Development of non-technical skills Challenges with non-verbal communication cues Authentic and common scenarios	*setting the right mindset of how to communicate effectively with clients and gather information while building rapport with them* (ST) *many aspects of body language are lost as mainly the face is seen online* (FA) *students did learn to communicate with clients in common scenarios* (FA)
3. Feedback and reflective practice	Receiving feedback from peers and facilitators Preparation and active engagement	*receiving feedback from other students and a tutor was really helpful; improved a lot between first and last workshop* (ST) *being given different cases each week and having time to prepare allowed me to be fully invested in the class and participate as actively as possible...get the most out of the sessions* (ST)
4. Workshop structure	Facilitator-led small groups GVCCM framework	*good to have small groups where instructors can give personal comments* (ST) *I think it works as a guide but I always stress the need for flexibility* (FA)
5. Ability and confidence	Progression Prior experience	*practice increases confidence; notable progression throughout the course* (FA) *the students that came with experience as a generalisation did the best (FA)*
6. Emotional response	Stress levels	*they were not so much stressful as much as they were engaging, enjoyable and fun, especially the assessment* (ST) *they didn’t seem unduly stressed. A bit of nerves, but that is good* (FA)

**Table 7:** *p*-values measuring associations between confidence levels and the number of opportunities to apply skills and the stage of degree.

Communication Skill	Number of Opportunities *p*-Value	Stage of Degree *p*-Value
Build rapport	0.125	0.938
Patient history	0.183	0.799
Client feels valued	**0.027**	0.889

**Table 8:** Predicted proportions for making a client feel valued based on the number of opportunities to apply skills.

	Predicted Proportion for “Yes”	Standard Error
No Opportunity	0.5	0.354
<10	0.307	0.128
>10	0.8	0.089

## Data Availability

The data presented in this study are available on reasonable request from the corresponding author.
